# A real-world study: third-line treatment options for metastatic colorectal cancer

**DOI:** 10.3389/fonc.2024.1480704

**Published:** 2024-12-02

**Authors:** Chen Wu, Shuai Li, Xinfang Hou

**Affiliations:** Department of Medical Oncology, Affiliated Cancer Hospital of Zhengzhou University, Henan Cancer Hospital, Zhengzhou, China

**Keywords:** colorectal cancer, targeted therapies, PD-1 inhibitors, bevacizumab, oncology

## Abstract

**Background:**

Numerous third-line treatment options exist for colorectal cancer. This study aims to assess the efficacy and safety of third-line therapies, including TKIs (fruquintinib, regorafenib) combined with PD-1 inhibitors, and trifluridine/tipiracil combined with bevacizumab, in patients with refractory microsatellite stable metastatic colorectal cancer who have progressed or are intolerant following standard first- and second-line treatments.

**Materials and methods:**

This retrospective analysis collected data from patients with microsatellite stable advanced colorectal adenocarcinoma, diagnosed through histopathology and treated at Henan Provincial Cancer Hospital from May 2019 to April 2023. We compared the efficacy and safety of fruquintinib combined with PD-1 inhibitors, regorafenib combined with PD-1 inhibitors, and trifluridine/tipiracil combined with bevacizumab.

**Results:**

Among 60 eligible patients with refractory microsatellite stable metastatic colorectal adenocarcinoma, 29 (48.3%) received fruquintinib combined with PD-1 inhibitors, 15 (25%) received regorafenib combined with PD-1 inhibitors, and 16 (26.7%) received trifluridine/tipiracil combined with bevacizumab. The average follow-up period was 12.6 months (ranging from 2.3 to 37.6 months). After third-line treatment, the overall objective response rate (ORR) was 8.6%, and the disease control rate (DCR) was 78.6%. The median overall survival (OS) for the regorafenib, fruquintinib, and trifluridine/tipiracil groups was 19.2 months, 14.0 months, and 16.2 months, respectively, with no statistically significant differences observed. However, there were statistically significant differences in progression-free survival (PFS); the median PFS for the regorafenib group was 6.3 months, for the fruquintinib group was 4.2 months, and for the trifluridine/tipiracil group was 5.4 months. Pairwise comparisons indicated that the PFS for the regorafenib group was similar to that for the trifluridine/tipiracil group, both of which were superior to the fruquintinib group. Cox univariate regression analysis revealed that the presence of liver and peritoneal metastases was associated with PFS in third-line treatment.

**Conclusion:**

In the third-line treatment of colorectal cancer, regorafenib combined with PD-1 inhibitors and trifluridine/tipiracil combined with bevacizumab showed superiority over fruquintinib combined with PD-1 inhibitors in terms of PFS, but no statistically significant difference in OS was noted among the three groups.

## Introduction

Colorectal cancer (CRC), a malignant neoplasm, represents a significant global health challenge, with over 1.85 million new cases and 850,000 deaths annually, ranking it as the third leading cause of cancer mortality worldwide. Nearly 20% of CRC patients exhibit metastasis at the time of diagnosis, and metastatic CRC (mCRC) is often an incurable disease. Due to the high frequency of metastasis and drug resistance, colorectal cancer remains one of the most difficult cancers to treat despite all the advances in biological knowledge and treatment improvements (1–3). Approximately 70% to 75% of patients with metastatic colorectal cancer survive beyond one year, 30% to 35% beyond three years, and fewer than 20% beyond five years. The primary treatment for inoperable metastatic colorectal cancer involves systemic therapy, encompassing chemotherapy, targeted therapy, and immunotherapy. Colorectal cancer is caused by the activation of oncogene mutations and the inactivation of tumor suppressor genes, with the latter being the main cause (4, 5). Different genetic characteristics lead to different prognoses (6, 7). Genomic analyses, focusing on somatic mutations in genes such as KRAS, NRAS, and BRAF, aid in selecting targeted medications and predicting future survival outcomes. Based on numerous seminal studies, guidelines for patients with refractory metastatic colorectal adenocarcinoma that is microsatellite stable recommend a standard chemotherapy regimen of fluorouracil, oxaliplatin, and irinotecan for the first and second lines of treatment. This regimen is to be used in conjunction with targeted drugs against vascular endothelial growth factor (VEGF) and its receptor (VEGFR), and epidermal growth factor receptor (EGFR), such as bevacizumab or cetuximab, based on genetic testing (8). Regorafenib, a novel oral multi-kinase inhibitor, disrupts kinases involved in tumor angiogenesis (VEGFR1, VEGFR2, VEGFR3, TIE2), tumorigenesis (KIT, RET, RAF1, BRAF, and BRAFV600E), and the tumor microenvironment (PDGFR and FGFR) (9). According to the CORRECT study (10), regorafenib has been approved for treating metastatic colorectal cancer after failure of all standard therapies, marking it as the first small molecule multi-kinase inhibitor to demonstrate a survival advantage in this setting (11). Fruquintinib, a novel VEGFR inhibitor, has received approval from the China National Medical Products Administration for treating advanced colorectal cancer patients who have undergone at least two standard anticancer treatments, as evidenced by the FRESCO study (12). Trifluridine/tipiracil, an innovative oral antimetabolic agent comprising trifluridine (a thymidine nucleoside analog) and tipiracil (a potent thymidine phosphorylase inhibitor), is currently approved for patients with refractory metastatic colorectal cancer after standard chemotherapy (13). An amalgamation of results from multiple related meta-analyses indicates that the differences in overall survival (OS) and progression-free survival (PFS) among these three drugs in third-line treatment are generally insignificant (14–16). According to the C-TASK-FORCE study and Danish studies, trifluridine/tipiracil combined with bevacizumab has demonstrated promising activity and manageable safety (17, 18). In metastatic colorectal cancer, the clinical efficacy of anti-PD-1 therapy is typically limited to tumors with high microsatellite instability (MSI-H), which constitute only 4-6% of cases (19). However, the significant progress of the REGONIVO study (20) have led to the increasing researchers trying to use the combination therapy of TKIs and PD-1 inhibitors, and has shown efficacy to a certain extent, indicating that the combination therapy of targeted and immune for advanced colorectal cancer may be about to enter a new era. Nonetheless, many ongoing studies on targeted and immune combination therapy remain placebo-controlled, without direct comparisons. Hence, selecting the optimal third-line combination treatment for advanced colorectal cancer post-standard treatment continues to be a key focus of research.

## Materials and methods

### Ethical approval and informed consent

The Ethics Committee of Henan Cancer Hospital reviewed and approved the study protocol, assigning it the ethical approval number: 2023-187-002. Given the study’s retrospective and non-interventional nature, the committee waived the requirement for informed consent.

### Patient population

This study is a retrospective cohort analysis. It collected clinical data from patients treated at Henan Cancer Hospital from May 2019 to April 2023, who were diagnosed with histopathologically confirmed microsatellite-stable advanced colorectal adenocarcinoma and who underwent third-line treatment. These individuals had previously received standard first- and second-line treatments, predominantly involving fluorouracil, irinotecan, oxaliplatin, bevacizumab, and cetuximab, until experiencing progression or intolerance. The patients were in generally good condition, with ECOG performance status scores of 0-2.

### Treatment regimen

Patients received third-line treatment with either fruquintinib (Elunate, Hutchison Whampoa Pharmaceuticals) combined with a PD-1 inhibitor, regorafenib (Stivarga, Bayer) combined with a PD-1 inhibitor, or trifluridine/tipiracil (Lonsurf, Taiho Pharmaceutical Co., Ltd.; Suhoo, Qilu Pharmaceutical; Qilu, Chia Tai Tianqing) combined with bevacizumab (Avastin, Roche Pharma (Switzerland) Ltd.; Eritu, Jiangsu Hengrui Medicine Co., Ltd.; Ankada, Qilu Pharmaceutical; Dayutong, Cinda Biopharmaceutical). PD-1 inhibitors include, but are not limited to, sintilimab (Tyvyt, Innovent Biologics), camrelizumab (AiRuiKa, Jiangsu Hengrui Medicine Co., Ltd.), tislelizumab (BaiZeAn, BeiGene Ltd.), toripalimab (Tuoyi, Suzhou Zelgen Biopharmaceuticals), nivolumab (Opdivo, Bristol-Myers Squibb), and pembrolizumab (Keytruda, Merck), all of which were administered in accordance with routine clinical treatment protocols.

### Efficacy and safety

Patients were monitored according to the routine protocols of Henan Cancer Hospital, with survival data obtained from the hospital’s follow-up center through April 28, 2023. Clinical efficacy was assessed through imaging examinations according to the Response Evaluation Criteria in Solid Tumors (RECIST version 1.1), encompassing complete response (CR), partial response (PR), stable disease (SD), and progressive disease (PD). The objective response rate (ORR) is calculated as the sum of CR and PR, while the disease control rate (DCR) includes CR, PR, and SD. The Common Terminology Criteria for Adverse Events (version 5.0) were utilized to evaluate drug toxicity. Progression-free survival (PFS) was determined from the initiation of combined treatment to disease progression (either clinical or radiological) or death from any cause, whichever came first. Overall survival (OS) was calculated from the beginning of treatment until death from any cause.

### Statistical analysis

PFS is characterized as the duration from the onset of third-line treatment to disease progression or death, while OS refers to the time from the start of third-line treatment to the patient’s death or the last follow-up. The Kaplan-Meier method is employed for survival analysis. The Pearson’s chi-square test or Fisher’s exact test are applied to evaluate differences between groups. Univariate analysis identifies predictive factors for PFS and OS. Statistical analysis and visualization are conducted using SPSS 27 and PRISM 9.0. The significance level (α) is established at 0.05.

## Results

### Patient characteristics and treatment

Based on the inclusion and exclusion criteria, clinical data from 60 patients with microsatellite-stable metastatic colorectal adenocarcinoma who underwent third-line treatment were analyzed. **Table 1** presents the demographic characteristics, disease status, and treatment details of these patients. The median age was 56 years, ranging from 31 to 75 years, with a majority (56.7%) being male. The rectum and sigmoid colon were the most common sites of the primary tumor, with 36 patients (60.0%) having tumors in the rectum and 9 patients (15.0%) in the sigmoid colon. A significant proportion of patients (51.7%) were diagnosed at stage IV. During the third-line treatment period, 12 patients (20.0%) received local combined therapy, including local radiotherapy, particle implantation, interventional embolization, and local surgery. Gene mutations were prevalent among the patients, with 37 individuals (61.7%) exhibiting KRAS mutations. Treatment regimens varied; 29 patients (48.3%) received fruquintinib combined with a PD-1 inhibitor, surpassing the numbers treated with regorafenib combined with a PD-1 inhibitor (15 patients, 25.0%) and trifluridine/tipiracil combined with bevacizumab (16 patients, 26.7%).

**Table 1:** Demographic and baseline characteristics.

		N=60
Gender (%)		
	Male	34 (56.7)
	Female	26 (43.3)
Age (%)		
	Over 60 years	22 (36.7)
	60 years or under	38 (63.3)
Primary Tumor Location (%)		
	Rectum	36 (60.0)
	Sigmoid colon	9 (15.0)
	Descending colon	2 (3.3)
	Transverse colon	5 (8.3)
	Ascending colon	6 (10.0)
	Cecum	1 (1.7)
	Hepatic flexure	1 (1.7)
Stage at Diagnosis (%)		
	I	4 (6.7)
	II	4 (6.7)
	III	19 (31.7)
	IV	31 (51.7)
	Unknown	2 (3.3)
Location of Metastases (%)		
	Single organ	14 (23.3)
	Multiple organs	46 (76.7)
Genetic Type (%)		
	All wild-type	21 (35.0)
	KRAS mutation	37 (61.7)
	NRAS mutation	2 (3.3)
Combined Local Treatment (%)		
	Yes	12 (20.0)
	No	48 (80.0)
Treatment Regimen (%)		
	Fruquintinib combined with PD-1 inhibitor Fruquintinib combined with a PD-1 inhibitor	29 (48.3)
	Regorafenib combined with PD-1 inhibitor	15 (25.0)
	Trifluridine/tipiracil combined with bevacizumab	16 (26.7)
Previous Surgery (%)	Curative surgery	33 (55.0)
	Palliative surgery	4 (6.7)
	No surgery	23 (38.3)

### Efficacy

**Figure 1:**
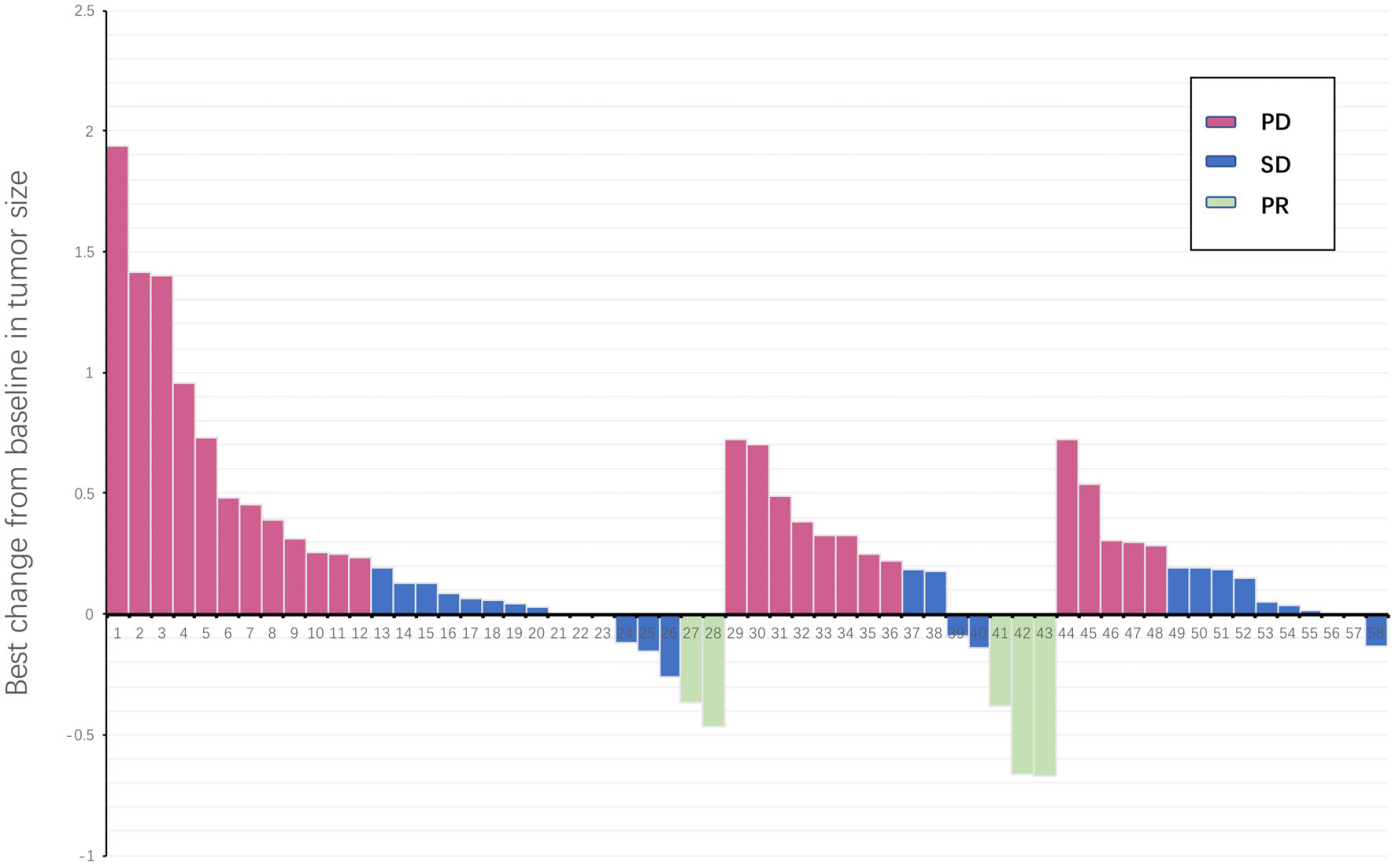
Waterfall plot illustrating maximum change in target lesion size for all treatment line patients (N = 58). The horizontal axis represents all patients included in the study, and the vertical axis represents the proportion of tumor size changes in patients.  PD, progressive disease; PR, partial response; SD, stable disease.

The average follow-up period was 12.6 months (2.3-37.6 months). Following third-line treatment, 2 patients did not provide evaluation results, 0 patients achieved CR, 5 patients achieved PR, 32 patients exhibited SD, and 21 patients experienced PD. Within the regorafenib combination group, there were 3 PRs, 5 SDs, and 7 PDs; in the fruquintinib combination group, there were 2 PRs, 16 SDs, and 10 PDs; and in the trifluridine/tipiracil combination group, there were 11 SDs and 3 PDs (**Table 2**; **Figure 1**). The overall objective response rate (ORR) was 8.6%, and the disease control rate (DCR) was 63.8%. Specifically, the ORR and DCR in the regorafenib combination group were 20% and 53.3%, respectively; 6.9% and 62.1% in the fruquintinib combination group; and 0% and 78.6% in the trifluridine/tipiracil combination group.

**Table 2:** The antitumor response of each treatment group assessed by response evaluation criteria in solid tumor Version 1.1.

Response Rates N (%)	Regorafenib	Fruquintinib	Trifluridine/Tipiracil	*p*-Value
**CR**	0 (0)	0 (0)	0 (0)	
**PR**	3 (5.2)	2 (3.4)	0 (0)	0.143
**SD**	5 (8.6)	16 (27.6)	11 (19.0)	0.05
**PD**	7 (12.1)	11 (19.0)	3 (5.2)	0.355

**Figure 2:**
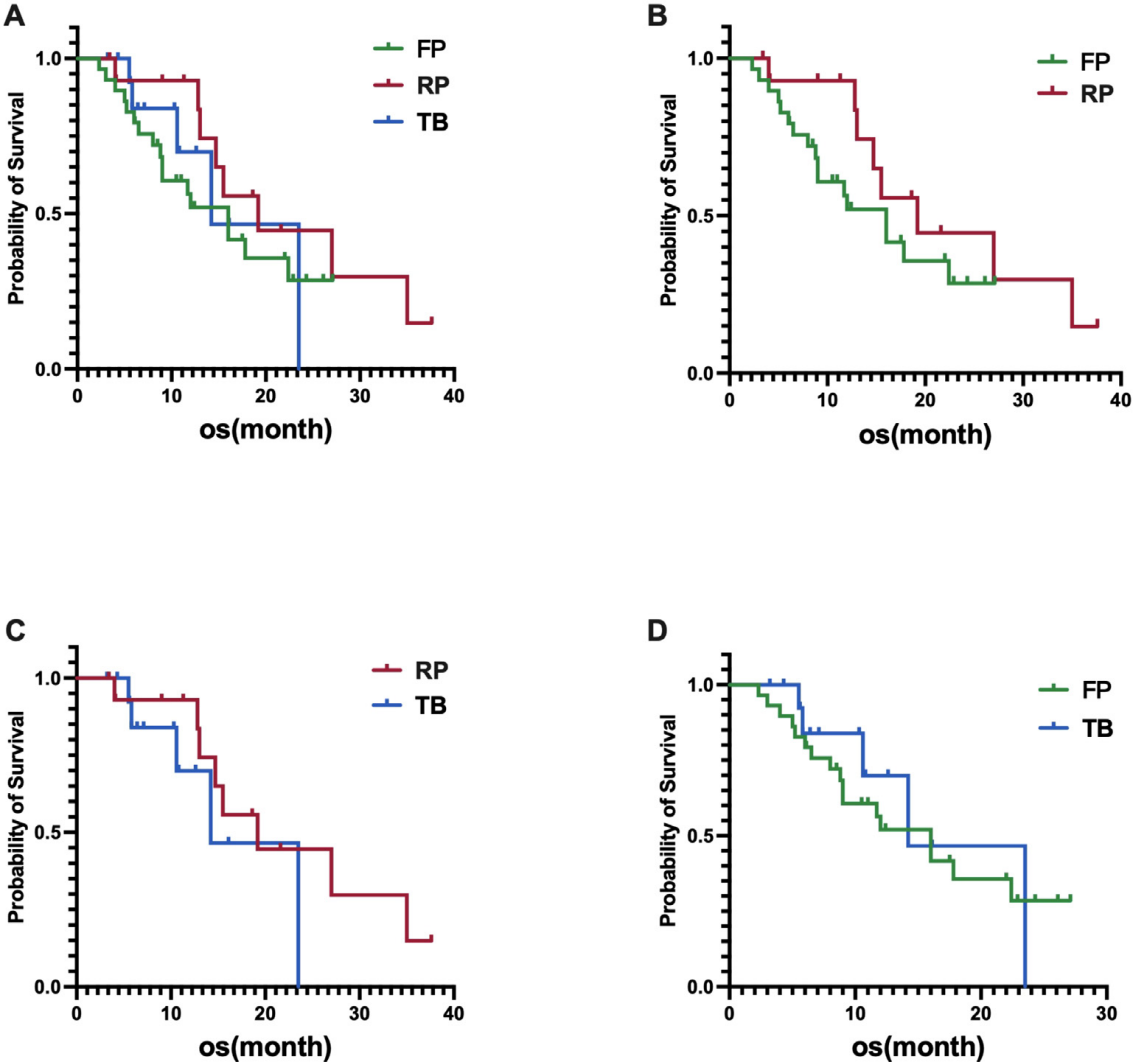
**(A)** Difference in OS among the three groups; **(B)** Difference in OS between FP (fruquintinib plus PD-1 inhibitor) and RP (regorafenib plus PD-1 inhibitor); **(C)** Difference in OS between RP (regorafenib plus PD-1 inhibitor) and TB (trifluridine/tipiracil plus bevacizumab); **(D)** Difference in OS between FP (fruquintinib plus PD-1 inhibitor) and TB (trifluridine/tipiracil plus bevacizumab).

In the fruquintinib group, the median OS was 16.0 months (95% CI, 10.2-21.8 months), in the regorafenib group, the median OS was 19.2 months (95% CI, 9.1-29.3 months), and in the trifluridine/tipiracil group, the median OS was 14.2 months (95% CI, 6.4-22.0 months). Statistical analysis revealed no significant differences in OS among the three groups, with a p-value of 0.4814 (**Figure 2A**). Similarly, no significant differences in OS were observed between the fruquintinib and regorafenib groups, with a p-value of 0.2475(HR 0.61) (**Figure 2B**); between the regorafenib and trifluridine/tipiracil groups, with a p-value of 0.2994 (HR 0.50)(**Figure 2C**); and between the fruquintinib and trifluridine/tipiracil groups, with a p-value of 0.6646 (HR 0.89)(**Figure 2D**).

**Figure 3:**
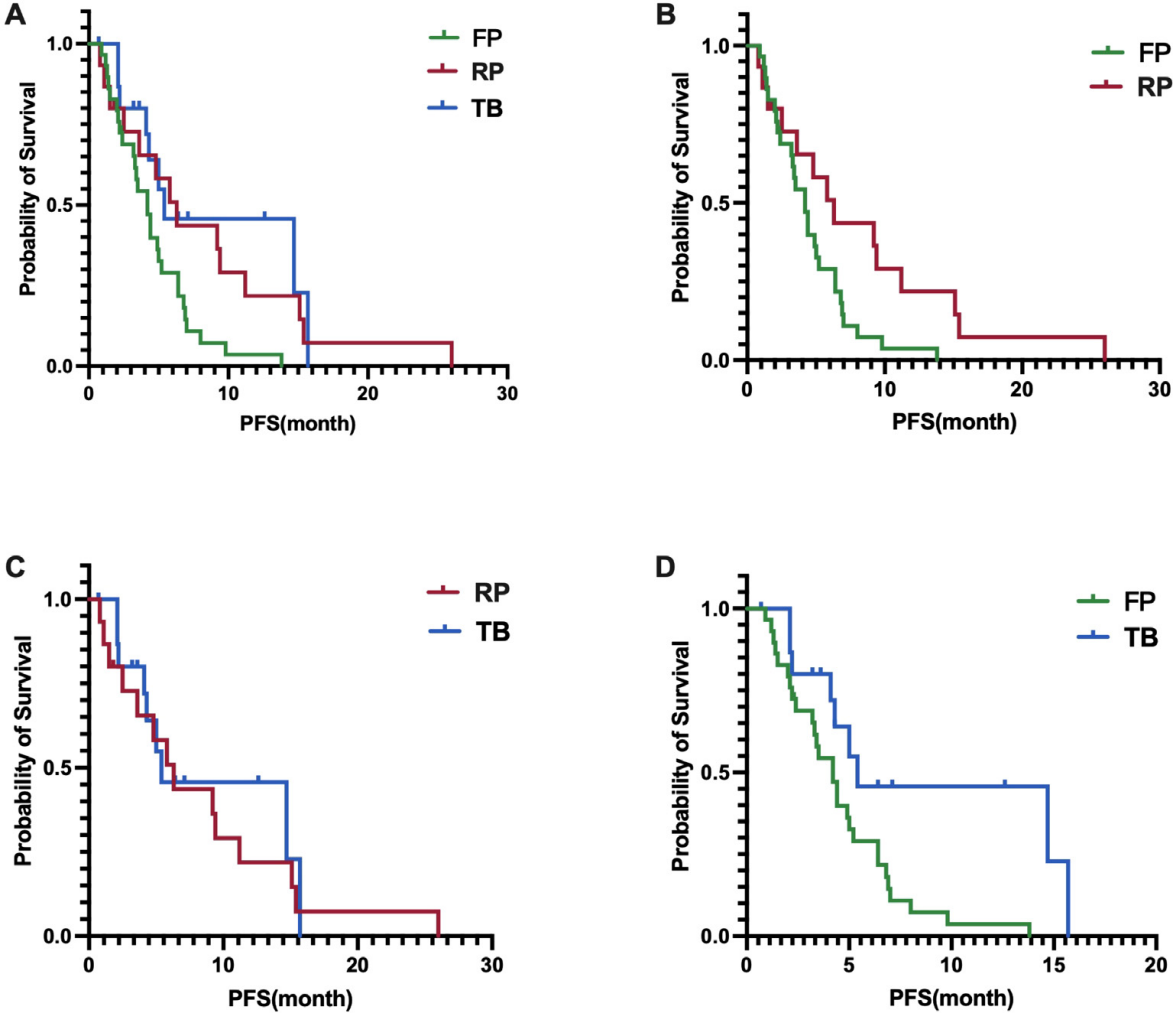
**(A)** Difference in PFS among the three groups; **(B)** Difference in PFS between FP and RP; **(C)** Difference in PFS between RP and TB; **(D)** Difference in PFS between FP and TB.

In terms of PFS, the fruquintinib group had a median PFS of 4.2 months (95% CI, 3.2-5.2 months), the regorafenib group had a median PFS of 6.3 months (95% CI, 3.6-9.0 months), and the trifluridine/tipiracil group had a median PFS of 5.4 months (95% CI, 0-12.5 months). A statistically significant difference in PFS was found among the three groups, with a p-value of 0.0157 (**Figure 3A**). A significant difference in PFS was noted between the fruquintinib and regorafenib groups, with a p-value of 0.0316 (HR 0.48)(**Figure 3B**); however, no significant difference in PFS was found between the regorafenib and trifluridine/tipiracil groups, with a p-value of 0.6402(HR 0.82) (**Figure 3C**); and a significant difference in PFS was observed between the fruquintinib and trifluridine/tipiracil groups, with a p-value of 0.0113(HR 0.41) (**Figure 3D**).

### Factor analysis

**Figure 4:**
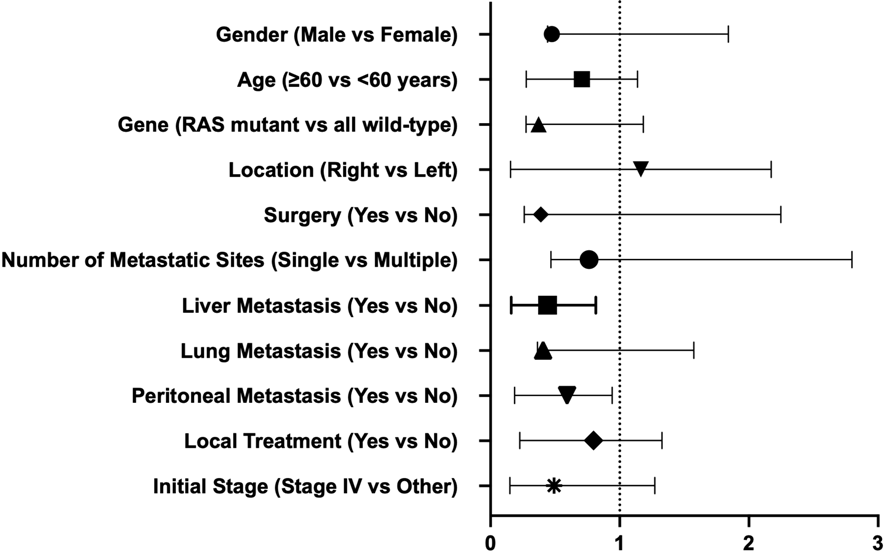
Univariate COX regression to determine factors associated with PFS.

Univariate Cox regression analysis indicated that for all participants, OS was not associated with baseline characteristics (age, gender, lesion location, stage at diagnosis, genotype, number of metastatic sites, and the presence or absence of liver, lung, and peritoneal metastases, combined local treatment, and surgery) (**Table 3**). However, the presence of liver and peritoneal metastases was associated with PFS in third-line treatment, while age, gender, lesion location, stage at diagnosis, genotype, number of metastatic sites, the presence or absence of lung metastasis, combined local treatment, and surgery were not associated with PFS (**Figure 4**).

**Table 3:** Univariate COX regression to determine factors associated with PFS and OS.

	PFS		OS	
	HR(95%CI)	*p-*Value	HR(95%CI)	*p-*Value
**Gender (Male vs Female)**	0.441 (0.475-1.842)	0.847	0.567 (0.768-0.4389)	0.172
**Age (≥60 vs <60 years)**	0.644 (0.312-1.167)	0.133	0.367 (0.366-2.207)	0.816
**Gene (RAS mutant vs all wild-type)**	0.373 (0.278-1.184)	0.132	0.633 (0.412-2.367)	0.976
**Location (Right vs Left)**	0.797 (0.391-2.305)	0.908	0.217 (0.376-3.634)	0.788
**Surgery (Yes vs No)**	0.39 (0.262-2.247)	0.629	0.617(0.182-2.98)	0.668
**Number of Metastatic Sites (Single vs Multiple)**	0.763 (0.468-2.797)	0.768	0.233(0.214-2.174)	0.517
**Liver Metastasis (Yes vs No)**	0.441 (0.161-0.815)	0.014	0.55(0.607-4.151)	0.346
**Lung Metastasis (Yes vs No)**	0.407 (0.364-1.574)	0.456	0.583(0.254-1.623)	0.349
**Peritoneal Metastasis (Yes vs No)**	0.593 (0.186-0.942)	0.035	0.417(0.356-2.634)	0.949
**Local Treatment (Yes vs No)**	0.797 (0.227-1.328)	0.183	0.2(0.391-3.166)	0.841
**Initial Stage (Stage IV vs Other)**	0.492 (0.151-1.271)	0.129	0.517(0.428-7.782)	0.416

### Safety

Of all participants, twenty-eight patients reported treatment-related adverse events (TRAEs), with four cases (14.3%) experiencing grade 3-4 TRAEs, including two cases of myelosuppression, one case of myocarditis, and one case of rash. Specifically, seven patients (25.0%) experienced myelosuppression, four (14.3%) reported poor appetite, five (17.9%) developed rashes, four (14.3%) experienced fatigue, three (10.7%) had myocarditis, four (14.3%) developed hypertension, two (7.1%) experienced vomiting, and there was one case (3.2%) each of incomplete intestinal obstruction, abdominal pain, abdominal distension, intestinal fistula, vaginal bleeding, ventricular premature beats, chest pain, and abnormal transaminase levels.

Among patients receiving fruquintinib combination therapy, 14 reported treatment-related adverse events, with no grade 3-4 TRAEs observed. Adverse events included hypertension (two patients), poor appetite (three), rashes (two), fatigue (three), and myocarditis (two). In the regorafenib combination therapy group, seven patients experienced treatment-related adverse events, including two cases of grade 3-4 TRAEs (myocarditis and rash), along with three instances of rash and two of hypertension. For those undergoing trifluridine/tipiracil combination therapy, seven reported treatment-related adverse events, with two experiencing grade 3-4 TRAEs (grade 4 myelosuppression); the remainder comprised myelosuppression (five cases) and vomiting (two cases). Clinicians successfully managed all adverse events, resulting in no TRAE-related fatalities (**Table 4**).

**Table 4:** Treatment-related adverse reactions.

	N=28 (%)	RP	FP	TB	*p*-Value
**Grade 3-4 Events**	4 (14.3)	2	0	2	0.134
**Myelosuppression**	7 (25.0)	0	2	5	0.014
**Poor Appetite**	4 (14.3)	0	3	1	0.426
**Rash**	5 (17.9)	3	2	0	0.122
**Fatigue**	3 (10.7)	0	3	0	0.185
**Hypertension**	4 (14.3)	2	2	0	0.330
**Myocarditis**	3 (10.7)	1	2	0	0.563
**Vomiting**	2 (7.1)	0	1	2	0.243
**Incomplete Intestinal Obstruction**	1 (3.6)	0	1	0	0.581
**Intestinal Fistula**	1 (3.6)	0	1	0	0.581
**Abdominal Distension**	1 (3.6)	1	0	0	0.218
**Vaginal Bleeding**	1 (3.6)	0	1	0	0.581
**Ventricular Premature Beats**	1 (3.6)	0	1	0	0.581
**Chest Pain**	1 (3.6)	1	0	0	0.218
**Abnormal Transaminase**	1 (3.6)	0	1	0	0.581

## Discussion

Currently, the third-line treatment for microsatellite stabilized colorectal adenocarcinoma includes multiple options, including chemotherapy and targeted therapy (8, 21). Cetuximab and bevacizumab have been received since most are in first - and second-line standard therapy. Based on CSCO guidelines in China and relevant studies, third-line therapies typically include rifenib, fruquininib and trifluridine/tipiracil monotherapy and trifluridine/tipiracil and bevacizumab combination therapy (21). For example, an open-label, randomized Phase II trial involving advanced colorectal cancer patients from four Danish centers, who had not responded to standard treatments, demonstrated that the combination of trifluridine/tipiracil and bevacizumab achieved a median PFS of 4.6 months, compared to 2.6 months for trifluridine/tipiracil alone (p=0.0015) (17). The REGONIVO study further revealed that regorafenib combined with a PD-1 inhibitor in advanced MSS colorectal cancer patients yielded an ORR of 33%, significantly influencing patient treatment and underscoring the effective synergy between PD-1 inhibitors and TKIs in this cancer type. Although the REGONIVO study had advanced the development of immunocombination targeting in colon cancer, it was not recommended in the guidelines because follow-up studies have failed to replicate the previous superior efficacy. Subsequent investigations reported that combining fruquintinib with PD-1 inhibitors led to a median PFS of 3.8-6.4 months, median OS of 11.1-14.9 months, ORR of 7.1%-21.05%, and DCR of 62.2%-89.3% (22–25), signifying an improvement from the median OS of 9.3 months and PFS of 3.7 months observed with fruquintinib monotherapy in mCRC patients as outlined in the FRESCO-2 study (26). In the pMMR population, with the combination of regorafenib and PD-1 inhibitors, PFS ranged from 4.0-7.9 months, OS from 11.1-15.03 months, and the highest DCR reached 70.8% (27, 28). Nevertheless, in the realm of third-line treatment for advanced MSS colorectal cancer patients, there remains a notable absence of direct comparison studies to more effectively guide the selection of combination therapies.

In our study, the fruquintinib combination group exhibited an ORR of 6.9%, a DCR of 62.1%, a median OS of 16.0 months, and a median PFS of 4.2 months. These outcomes are in line with those reported by Sun et al. (24) for the fruquintinib plus PD-1 inhibitor group in terms of ORR and DCR and are comparable to the DCR noted by GOU et al. (23), albeit with a marginally lower ORR. The regorafenib combination group demonstrated an ORR of 20%, DCR of 53.3%, median OS of 19.2 months, and median PFS of 6.3 months. These figures surpass the ORR of 7.1% reported by Sun et al. (24) and align closely with their reported DCR of 56.5%; both the OS and PFS exceed the findings from Chen et al.’s study (27) on elderly colorectal cancer patients. Despite regorafenib and fruquintinib both being oral anti-angiogenesis medications, regorafenib’s multi-target capabilities contrast with fruquintinib’s high selectivity for VEGFR1, VEGFR2, and VEGFR3 (29), potentially explaining the varied treatment outcomes. Nonetheless, the ORR and PFS for TKI drugs combined with immunotherapy were lower than the 33% ORR and 7.9 months PFS observed in the REGONIVO study (20). This discrepancy may be attributed to the phase 1b nature of the REGONIVO trial, which focused on dose exploration and expansion to establish safety and recommend dosages. Additionally, the REGONIVO study’s results, primarily from Japan, might reflect ethnic differences and distinct disease management practices, contributing to the variation in outcomes. The diverse PD-1 inhibitors used in combination could also influence the results. In addition, due to the small sample size of our study, there may be biases. In our study, patients had prolonged OS compared to other studies. We considered the following aspects: First, because the retrospective analysis did not exclude patients receiving local treatment, some patients may benefit from local treatment; Secondly, whether it is related to the long-term benefits obtained by some patients from immunotherapy needs further exploration and research. In addition, some patients with good ECOG score received 1-2 lines of follow-up treatment, and some may benefit from follow-up treatment. Finally, due to our limited sample size and the limitations of retrospective study follow-up, there are some deleted data, which may affect the OS data due to follow-up. In our analysis, the trifluridine/tipiracil combination group recorded an ORR of 0%, DCR of 78.6%, median OS of 14.2 months, and median PFS of 5.4 months. These results surpass those of studies such as TAS-CC3 and TAS-CC4 (25, 30, 31) in terms of OS and PFS, which may be due to racial differences, the employment of biweekly versus weekly dosing schedules, and our adoption of a three-week regimen. Furthermore, clinical trials commonly set Eastern Cooperative Oncology Group (ECOG) performance scores at 0-1, a standard challenging to rigorously maintain in clinical practice; these were phase II studies, necessitating further validation. Although there were patients with both wild-type and mutant RAS in our study, due to the relatively standardized early treatment, treatment options such as the rechallenge of cetuxib were not considered in third-line treatment, and the type of RAS had nothing to do with the benefits of third-line treatment options.

Regarding adverse reactions, our study documented a 14.3% incidence rate of grade 3-4 adverse events, with two instances of myelosuppression identified in the trifluridine/tipiracil group and immunological inflammation and rashes predominantly observed in the TKI plus PD-1 inhibitor groups. Overall, the incidence rate of myelosuppression exhibited variation among the three groups, while other adverse reactions were not significantly different, aligning with findings from other studies. This suggests that the tolerance for the combined treatments in advanced colorectal adenocarcinoma is generally favorable, with manageable adverse reactions. Upon reviewing the patients’ baseline conditions, we discovered that peritoneal or liver metastasis could impact PFS; however, the limited number of participants underscores the necessity for further investigation with a larger cohort.

In summary, our study represents the first comparative analysis of combination treatments involving regorafenib, fruquintinib, and trifluridine/tipiracil. The findings suggest that, with respect to PFS, both the regorafenib and trifluridine/tipiracil groups surpassed the fruquintinib group, yet there were no significant differences in OS among the three groups. A more holistic approach to selecting third-line treatment options should incorporate economic considerations and the specific needs of the patient. Considering our study’s retrospective nature and the limited sample size, the efficacy and safety of these treatment options require further clinical investigation in the future.

## Conclusion

In this third-line treatment for colorectal cancer in the real world, although rifenil combined with PD-1 inhibitor and trefludine/tipirizumab combined with bevacizumab were superior to fruquintinib combined with PD-1 inhibitor in PFS, there was no significant difference in OS among the three groups. In third-line treatments, prospective studies are needed to confirm current findings and make better choices.

## Data Availability

The raw data supporting the conclusions of this article will be made available by the authors, without undue reservation.
